# Psychological Wellbeing, Mindfulness, and Immunity of Teachers in Second or Foreign Language Education: A Theoretical Review

**DOI:** 10.3389/fpsyg.2021.720340

**Published:** 2021-07-15

**Authors:** Shengji Li

**Affiliations:** Ural Institute of North China University of Water Resources and Electric Power, Zhengzhou, China

**Keywords:** L2 education, positive psychology, psychological well-being, mindfulness, teacher immunity

## Abstract

The emotions and affective factors of teachers play quintessential roles in academic contexts as they influence almost all aspects of their profession. T provide a theoretical review of some psychology constructs of teachers in this area, this study examines the psychological wellbeing, mindfulness, and immunity of teachers as three novel variables. More specifically, this study presents the definitions, dimensions, theories, and frameworks related to this domain drawing on positive psychology, complexity/dynamic systems theory, self-organization process, reflexive self-consciousness theory, integrative awareness theories, and mindfulness framework. These theoretical underpinnings explain the constructs and the way they function in second language education. Then, to provide evidence and justify the findings and propositions, empirical studies on each of the variables are reviewed. Finally, implications, research gaps, and suggestions for future inquiries are offered to interested researchers.

## Introduction

Teaching has long been endorsed to be one of the most demanding and stressful jobs with high levels of tension, burnout, attrition, and low professional wellbeing (Benevene et al., 2020; Mercer, 2020). In the context of second/foreign language education, the story gets more complicated in the sense that teachers should, additionally, grapple with linguistic, intercultural, and pedagogical difficulties of their job (Gkonou and Miller, 2017; King and Ng, 2018; Nayernia and Babayan, 2019). That is why teachers are regarded as the most valuable, costly, and “critical pillars” of academia (Khani and Mirzaee, 2015; Pishghadam et al., 2021), whose emotions and needs must be put at the top of research and educational agendas (Maslach and Leiter, 1999; Pishghadam et al., 2019; Mercer, 2020). With the advent of this shifting trend toward “the psychology of teachers,” the myriads of research studies have been conducted on different aspects of language teaching and their impacts on the achievement of learners. However, a great majority of such teacher-related investigations have capitalized on the negative factors, such as stress, exhaustion, and fatigue, all leading to non-functionality in teachers (Fleming et al., 2013; Benevene et al., 2020; Jin et al., 2020). Research on these stressors pinpointed that the health, job satisfaction, identity, self-efficacy, efficiency, motivation, and professional performance of teachers are all reliant on the degree of care that educational institutions pay to their frontline soldiers (Derakhshan et al., 2019, 2020).

Nevertheless, with the emergence of positive psychology (PP) as a new school of psychology that was the offshoot of humanism, scholars turned their attention from negative to positive sides of teaching and explored the emotions, care, wellbeing, and credibility of teachers (Jin et al., 2020; Pishghadam et al., 2021). Simply, PP is an approach that examines how people thrive and try to lead happier lives (MacIntyre and Mercer, 2014; MacIntyre et al., 2019; MacIntyre, 2021). It highlights the roles that courage, optimism, resilience, hope, happiness, flow, creativity, interpersonal communication skills, credibility, clarity, wellbeing, and others play in academia (Seligman and Csikszentmihalyi, 2014).

As a construct that is best observable in PP, the psychological wellbeing (PWB) of teachers is described as the judgment and satisfaction of an individual with his/her happiness, physical and mental health, and profession (Huppert, 2009). The wellbeing of teachers goes beyond the simple absence of setbacks and stressors at work and concerns healthy and functional teachers. In simple terms, wellbeing refers to the capability of teachers to strike a positive and dynamic balance between their resources and professional challenges (Benevene et al., 2020). It brings about different positive outcomes in the academic arena such as forming a positive rapport among teachers and students, promoting the didactic performance of teachers, raising job satisfaction level, and increasing the achievement level of students (Roffey, 2012; Kidger et al., 2016; Fathi and Derakhshan, 2019). In foreign language contexts in which teachers have to fight in two arenas (i.e., transferring knowledge and removing barriers), there is an urgent need to take into consideration the PWB of teachers in institutions whose structure and climate determine the level of PWB (Tang et al., 2013). At present, the emotions and psychological attributes of teachers play a significant role in teaching creatively and effectively (Roffey, 2012). They perform best in an environment in which their affective, professional, and social needs are fulfilled. Similar to other aspects, research shows that the PWB and academic performance of students are largely dependent on the PWB of teachers (Briner and Dewberry, 2007; Bentea, 2015). Therefore, it is crucial for educational institutions to pay special attention to the PWB of teachers if they wish to have high-quality teaching and ultimately improved student learning.

As a concept highly related to the PWB of teachers, which reflects the complexities of the teaching profession, mindfulness has been derived from Buddhism to describe living with awareness, which needs effort and training (Siegel, 2007; Hassed and Chambers, 2015). It is also considered as an intrinsic process that stresses his/her awareness of both internal and external experiences as they happen (Brown and Ryan, 2003). As stated by Rix and Bernay (2014), mindfulness is the full attention of an individual on what is occurring inside and nearby him/her both physically and mentally. The concept is important in the second/foreign language education context in which teachers in this job are pressurized from different angles such as emotional-affective sides and environmental–professional sides. This is a daunting task in the second language (L2) education as teachers in this setting have to deal with many issues at the same time. All such attentional stimuli place stress, self-doubt, and tension on teachers which mostly end in burnout and job-leaving (Waldman and Carmel, 2019; Li et al., 2021). The construct of mindfulness has been identified to promote and correlate positively with different variables in education such as wellbeing (Hue and Lau, 2015), academic performance (Rosenstreich and Margalit, 2015), task engagement (Kee and Liu, 2011), emotion regulation (Kerr et al., 2017), and teaching effectiveness (Flook et al., 2013). Moreover, the mindfulness of teachers has been claimed to reduce their degree of weariness, stress, and anxiety (Regehr et al., 2013; Harnett et al., 2016).

Another novel construct that mirrors the complications of L2 education is teacher immunity, proposed by Hiver and Dörnyei (2017). It is meaningful in language education in that teachers have to deal with numerous challenges in the class aside from pedagogical issues. As a result, these days, teachers as “architects of society” are no longer judged, recruited, and studied only based on their teaching abilities but their psychology and emotion (Hiver and Dörnyei, 2017; Haseli Songhori et al., 2018). They convey their beliefs, feelings, attitudes, value systems, and behaviors to students indirectly, which signify the importance of the psychology of teachers (Haseli Songhori et al., 2018). In such a tense and strained context, L2 teachers usually seek protections against stressors such as low income, limited facilities, poor institutions, and other challenges that lead to attrition and burnout. The quest for overcoming these barriers brought into educational jargon a fresh concept known as “teacher immunity” that refers to the defense mechanisms of teachers that are utilized to minimize the disturbances and damages posed on their motivation, identity, and practice (Hiver, 2017). Immunity is so strong that can influence almost everything that teachers do in their profession (Maghsoudi, 2021). This armoring system (i.e., immunity) is useful for offering deeper insights into the cognition, experiences, and professional identity of English as a foreign language (EFL) teachers (Hiver and Dörnyei, 2017). However, due to its recency, teacher immunity research is still in its embryonic stages and seeks further developments and explorations. At present, most of the studies conducted in this line of research have focused on unpacking different triggers and coping strategies of EFL teachers when facing difficulties. Nevertheless, investigating the reasons and processes behind teacher immunity requires further research. Consequently, in this review article, the researchers made an effort to demystify this novel construct which is highly associated with the emotions, positive affect, wellbeing, and mindfulness of teachers.

## Theoretical Background

### Positive Psychology

Positive psychology is a movement in psychology and education introduced by Seligman (2006) as a paradigm shift against student-centered pedagogy. It has its roots in humanistic psychology and aims to examine how people flourish through their emotions and feelings that increase the quality of life (Csikszentmihalyi and Nakamura, 2011; MacIntyre et al., 2019). Moreover, PP is a reaction against the dominance of cognitivist perspectives in second-language acquisition (SLA) that had blurred the role of emotions in L2 learning. In this theory, life is not seen only from abnormalities and adversities but instead from positive sides (Seligman, 2006) to help people live a happier life. In other words, while its focus is on wellbeing, it does not disregard barriers and setbacks. Instead, it tackles them from the angle of human strengths in place of weaknesses (Oxford, 2016). In PP, the role of courage, wellbeing, perseverance, flow, hope, resilience, optimism, creativity, happiness, and the like are deeply explored. As pinpointed by Seligman (2011), this approach has three core pillars of positive emotions (i.e., positive, internal, and subjective experiences), positive character traits (i.e., features related to wellbeing), and positive institutions (i.e., workplaces that help an individual flourish). This shift of attention toward emotions and affective factors in education continued to gain popularity also in SLA. As a result, many studies were conducted on different constructs associated with this strand of research whose results pointed to the potentials of PP in improving the resilience, efficacy, immunity, credibility, clarity, motivation, engagement, creativity, and initiative of EFL teachers and students, and moderating the impact of negative emotions (Dewaele, 2015; Dewaele et al., 2019). However, as suggested by Li (2020), despite the significance of positive emotions in SLA, a majority of studies are still conducted around the globe to examine negative attributes, such as stress, anxiety, burnout, attrition, and self-doubt, and many other positive constructs in PP are left unexplored.

### Psychological Wellbeing

#### Conceptualization of Psychological Wellbeing

The notion of PWB is conceptualized as a process that entails many intertwined constructs and dimensions (Weiss et al., 2016). It has been drawn from PP and concerns his/her positive functioning, happiness, personal growth, flourishing the self, and so forth (Zaki, 2018). In their seminal attempt to make the concept more vivid, Ryan and Deci (2001) proposed two approaches to wellbeing, namely, “hedonic” and “eudemonic.” Hedonic approaches are only concerned with happiness and life satisfaction and describe wellbeing as obtaining pleasure and circumventing pain. The main purpose of hedonic approaches is to raise happiness forming what came to be known as “subjective wellbeing.” In contrast, eudemonic approaches to wellbeing highlight meaning and self-actualization and concern the living of human life and the realization of his/her potential. This approach provides the basis for PWB and capitalizes on his/her ability to use personal resources and strengths in such a way that he/she can give meaning to life (Mercer, 2020). It is noteworthy that the concept of PWB is not an on-and-off process to be justified either by hedonic or by eudemonic approaches. In many cases, the construct should be interpreted through a mixture of both approaches together with a consideration of his/her positive affect and emotions (Jayawickreme et al., 2012).

Additionally, it is unwise to conceptualize PWB as an individual-subjective variable but a multifaceted variable that can be both subjective and objective. It is a social and context-sensitive construct dynamically shaped through an interplay between conditions, environments, actions, mental resources, and interpersonal relations (La Placa et al., 2013). This perspective stresses the active role of the person in constructing and designing his/her wellbeing. However, it does not reject the influence of sociocultural contexts and policies in this area. PWB has been substantiated to affect many aspects of the life and profession of individuals due to its tight connection with emotions and mentality. Due to this, successful educational systems are now offering their teachers mediation programs to make teachers aware of wellbeing and its effects on their practice, efficacy, motivation, identity, and the wellbeing of students.

#### Factors Influencing the Wellbeing of Teacher

Due to the inherent complexities in L2 education, the wellbeing of EFL teachers (i.e., physical and mental) is largely influenced by numerous factors whose consideration can help in assisting stakeholders to thrive and have better experiences in academic settings. Among numerous possible factors aside from demographic factors, research indicates that the wellbeing of teachers is strongly affected by linguistic and intercultural demands of the second/foreign language, linguistic capital of language educators, institutional contexts, the emotional connections of teachers with students, the emotional labor of the second/foreign language, classroom encounters, practical supports, working conditions, the prospects of promotion and development of teachers, job security, vocational motives, and prosocial attitudes (Target, 2003; Borg, 2006; Wickham, 2015; Gkonou and Miller, 2017; King and Ng, 2018; Nayernia and Babayan, 2019; Skinner et al., 2019; Greenier et al., 2021). Additionally, it is crucial to mention that the wellbeing of teachers is a broad construct, which is likely to be affected by factors other than the abovementioned depending on educational contexts.

#### Dimensions of the Psychological Wellbeing of Teacher

**FIGURE 1:**
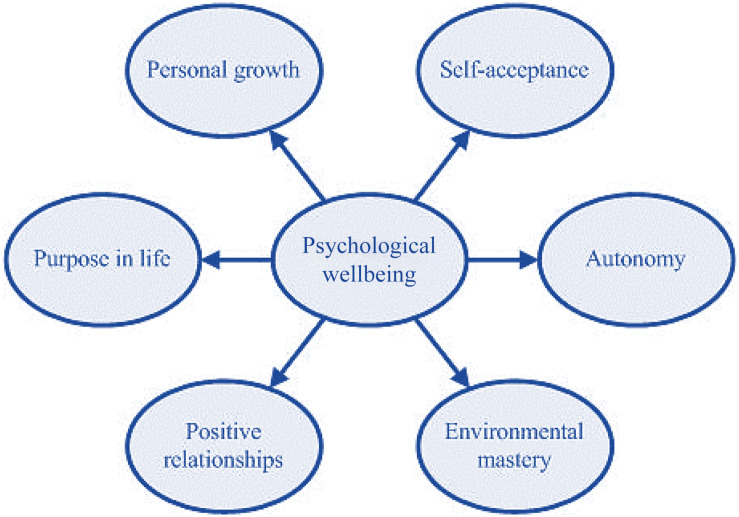
Dimensions of the psychological wellbeing (Ryff, 1989).

As stated earlier, with the advent of PP, scholars and practitioners shifted their attention toward positive emotions and affective factors in academia to provide an opportunity for teachers and students to have positive functioning (Zaki, 2018). This led to the emergence of PWB as a multidimensional construct in PP. According to the study of Ryff (1989), PWB is comprised of six dimensions, namely, *self-acceptance*, *positive relations with others*, *autonomy*, *personal growth*, *environmental mastery*, and *purpose in life* (Figure 1).

### Mindfulness

#### The Definition of Mindfulness

Over the past decades, different descriptions and interpretations have been provided by scholars of this domain for the concept of mindfulness. It has been regarded as a dynamic process internal to a human being, which focuses on his/her awareness of events and experiences as they occur (Brown and Ryan, 2003). It permits the brain to think, experience, and reflect on the current-moment experience and thoughts (Hölzel et al., 2011). Mindfulness is not a fixed variable but dynamic and changeable that can be (re)constructed in one through mediation and training (Roeser, 2016). Similarly, Roeser (2013) described mindfulness as a non-judgmental awareness of people regarding the present-time events that are interconnected with his/her tranquility and kindness. The concept has been used in clinical and educational contexts conveying a similar message. In clinical mediation contexts, mindfulness is a method to promote the awareness and ability of the patient to react appropriately to deficient behaviors such as anxiety and depression (Bishop et al., 2004). In educational settings, it refers to the process of training attention and regulating emotions in a way that an individual focuses only on the present moment without any regret and worry about past or future events (Ramasubramanian, 2017).

#### Theories and Frameworks of Mindfulness

Similar to its definitions, the “paraconceptual” construct of mindfulness has multiple theoretical frameworks (Brown and Ryan, 2003). Primarily, it belongs to the postpositivist tradition and constructivist paradigm, and its application in academia needs a harmony of different methodological perspectives (Creswell, 2014). Additionally, this novel construct has been inspired by and is in close association with different theories of attention and awareness. They include the *reflexive self-consciousness theory* that focuses on the person and his/her experiences as the object of attention (Duval and Wicklund, 1972), *integrative awareness theories* growing out of psychodynamic, humanistic, cognitive behavioral, and motivational orientations, which are interested in what is happening both internally and externally to the individual who increases his/her functioning (Ryan, 1995), and the *seminal theoretical framework* of Shapiro et al. (2006), which focuses on the dimensions of mindfulness through three main axioms, namely, *intention*, *attention*, and *attitude*. The first axiom refers to the intrinsic rationale behind practicing or desiring mindfulness. The second axiom concerns the attention paid to his/her moment-to-moment operations and experiences. The third axiom that directly affects the second axiom refers to how individuals perceive and approach the mindfulness practice (Shapiro et al., 2006). There are also other theories that lie under the mentioned theories such as Gestalt psychology, self-efficacy theory, self-regulation theory, flow theory, and self-determination theory whose presentation is beyond the space budget of this study.

### Immunity of Teacher: Definitions and Related Concepts

The notion of teacher immunity is derived from medicine referring to a line of defense or protective mechanisms that human uses to stand the challenges and adversities in his/her life or profession (Hiver, 2015; Rahmati et al., 2019). The term has been proposed by Hiver (2015) and Hiver and Dörnyei (2017) in their groundbreaking studies on existing gaps in the motivation and professional identity of teachers. Similar to the immune system of a human being, which fights against external germs and pathogens, the immune system of a teacher functions as an armoring mechanism to cope with setbacks and stressors in language teaching contexts that are full of adversities and complications (Hiver, 2017). In the academic arena, it provides a framework for explaining the processes through which language teachers find and use a defense system to remove or alleviate the impact of adversities on their motivation and professional identity (Hiver and Dörnyei, 2017).

Teacher immunity is recognized by some features such as *specificity*, *memory*, *adaptability*, and *durability*. Specificity means that teachers use specific strategies to deal with specific challenges/adversities. Memory refers to the use of past experiences of coping with tensions. Adaptability is his/her adjustability to change. Finally, durability denotes the permanency of protection until it becomes a part of his/her identity (Rahmati et al., 2019). Similar to medicines that cure and survive an organ but sometimes create allergies and incur damages to others, teacher immunity can also bring about negative outcomes. More specifically, teacher immunity can take two forms, namely, productive (i.e., positive) or maladaptive (i.e., negative). Teachers with productive immunity are not typically vulnerable to stress and failure and easily ignore conflicts and challenges, and therefore, they usually have high job satisfaction, self-confidence, and ultimately promote in their career. In contrast, teachers with maladaptive immunity have low confidence, self-esteem, motivation, and self-efficacy. As a result, they are extremely conservative in their instruction and resistant to any change (Hiver and Dörnyei, 2017).

Along with these conceptualizations, many cognate terms have also been proposed parallel to immunity such as *coping* (i.e., strategies employed to inhibit or get rid of stressors), *hardiness* (i.e., a personality trait that mitigates the psychological impacts of stress on his/her performance), *resilience* (i.e., the ability to recover from traumatic experiences or adversities), *adaptability* (i.e., the ability to adjust oneself to changing situations), and *buoyancy* (i.e., the self-perception of an individual of his/her ability to effectively manage encountered tensions, stressors, and challenging tasks) (Parker and Martin, 2009).

### The Theory Behind Teacher Immunity

The novel construct of teacher immunity is based on the complexity/dynamic systems theory (CDST) and the self-organization process as one of its core principles. CDST is regarded as a theory of change, evolution, and adaptation, which examines survival by a blend of cooperation and competition (Morrison, 2002). Based on this theory, there is always a dynamic and cyclical association between an organism and its surroundings, which often change each other (Battram, 1999). Moreover, the organism and its identity are subjected to modifications due to its networks and relationships in the context. The entire milieu and its parts work together dynamically and yield new realities and associations (Morrison, 2006). Teacher immunity is also explained through the self-organization process, which is the spontaneous pattern formation and change in CDST (Hiver, 2018). More specifically, the self-organization process is an adaptive process wherein the internal structure and function of a system vary in reaction to external surroundings to assure survival. This process has four developmental stages of *triggering*, *coupling*, *realignment*, and *stabilization* (Hiver, 2016). In the triggering stage, adversity takes place and the system loses its balance and becomes more susceptible than normal (Hiver and Dörnyei, 2017). In the coupling stage, through positive feedback loops, organisms interact with one another and the system and develop coping techniques to fight against adversities (Rahmati et al., 2019).

In realignment, the organism begins to restructure him/herself to gain stability (Thelen and Bates, 2003). Moreover, the system recaptures its productivity, and new behavioral patterns appear in the system. Finally, in the stabilization stage, as stability is reached, the organism shuns from susceptibility by defending itself against future conflicts. The new behavioral pattern is said to become a part of the behavior of the system and stabilizes in it (Rahmati et al., 2019). It is at this stage that teachers take productive or maladaptive forms of immunity.

## Empirical Studies

Research suggests that teachers are the builders of prosperous and successful societies, and to make learning possible, their emotions, needs, and psychological health must be assured in the academic contexts (Zaki, 2018). Due to the complexities and adversities involved in L2 education, language teachers are usually pressurized from many angles, this exerts tensions on them, and their instruction may lose effectiveness (Benevene et al., 2020). Becoming aware of the criticality of the emotions and affective factors of teachers, a movement emerged in psychology and education called PP whose aim is to explore positive emotions and interpersonal communication behaviors of teachers. In the past couple of decades, many studies have been conducted on different psychological factors of teachers such as teacher immediacy, care, clarity, credibility, motivation, engagement, and confirmation. This signifies a change of approach from negative factors such as the stress and anxiety of teachers to positive variables in this line of investigation.

As one of the most significant variables related to the psychology of teachers, the concept of PWB has recently witnessed a growing body of explorations in different contexts due to the rise in the job-leaving rate among teachers (Benevene et al., 2020). It has also been found that PWB helps in establishing a positive rapport between teachers and students (Fathi and Derakhshan, 2019), maximizing the academic performance of teachers (Fathi et al., 2020), increasing job satisfaction (Kidger et al., 2016), and increasing the achievement level of students (Bentea, 2015). Moreover, in contexts in which teachers enjoy a high level of PWB, the degree of stress, anxiety, and burnout is claimed to be kept at a minimum level (McCullough, 2015; Fathi and Saeedian, 2020). Similarly, in her recent study, Mercer (2020) took an ecological perspective to examine the wellbeing of English language teachers in Malta through the interpretative phenomenological analysis (IPA) approach and identified that the wellbeing of teachers is affected by the business model character, working conditions, and the status of teaching English as a job. Similarly, Zaki (2018) conducted a study on ways to promote the PWB of teachers in India and proposed that by increasing this construct, education and learning boost considerably. Furthermore, in Iran, Fathi et al. (2020) carried out a study on the association between PWB, self-efficacy, and collective efficacy using structural equation modeling (SEM) and found that both types of efficacy had a significant impact on the PWB of teachers with self-efficacy being slightly stronger. Research also shows that the PWB of teachers is highly influenced by negative factors, stressors, and burnout factors such as stress, tension, demotivation, depersonalization, and exhaustion (Schaufeli and Salanova, 2007). Hence, to help these constructs thrive, educational systems and institutions should eradicate these stressors and at the same time reinforce positive emotions in teachers.

As for mindfulness, Wang and Liu (2016) conducted a case study on 24 EFL students in China using surveys, observations, and journals and concluded that cultivating this variable leads students to become the agents of their learning process. Similarly, Chiesa et al. (2011) argued that through mindfulness practices and programs, the psychological potentials and regulation strategies of teachers increase substantially. It has also been found that the mindfulness of teachers expands their social relationship (Condon et al., 2013), self-care, and reflectivity beside the betterment of socio-academic circumstances for students to flourish (Shapiro et al., 2016). The correlational studies also pinpointed that mindfulness has close ties with the PWB of teachers (Jennings, 2015), the academic performance of teachers and students (Rosenstreich and Margalit, 2015), task engagement (Kee and Liu, 2011), emotional regulation (Kerr et al., 2017), and teaching effectiveness (Flook et al., 2013). Moreover, it has been found that mindfulness had a negative correlation with stress and anxiety (Harnett et al., 2016). Moreover, Waldman and Carmel (2019) took a practical approach and explored the effect of mindfulness on the self-efficacy of teachers to teach L2 writing in Israel. Using an experimental research design, they found that mindfulness practice for EFL teachers significantly increases their self-efficacy for teaching writing.

Concerning the last variable (i.e., immunity), which is affected by both teacher PWB and mindfulness, there is only a handful of research on it due to its novelty. As a case in point, in a landmark study, Hiver (2017) carried out a qualitative study in Korea to provide a universal classification for teacher immunity comprising as follows:

• Productively immunized teachers.
• Partially immunized teachers.
• Maladaptively immunized teachers.
• Partially maladaptively immunized teachers.
• Immune-compromised teachers (i.e., lack of immunity of teachers).

**FIGURE 2:**
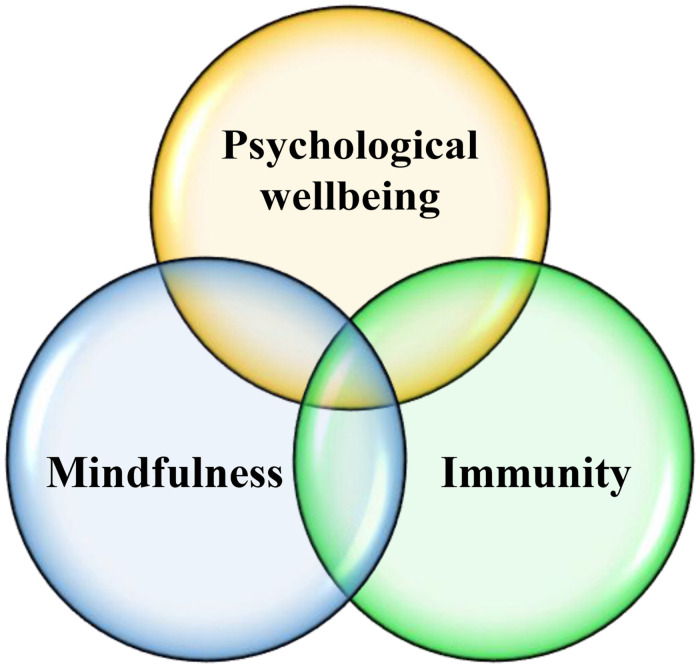
The relationship between the psychological wellbeing, mindfulness, and immunity of teachers.

Moreover, in their influential study, Hiver and Dörnyei (2017) maintained that teacher immunity is strongly correlated with professional survival, pedagogical practice, motivation, and different identity types of L2 teachers. Similarly, Saydam (2019) conducted a mixed-methods study on the immunity of 187 Turkish EFL teachers and found that most of the participants were productively and maladaptively immunized and their overall immunity level was high. In the EFL context of Iran, Haseli Songhori et al. (2018) employed a mixed-methods design to unpack the dominant immunity type of Iranian teachers. They handed a questionnaire to 230 EFL teachers followed by an interview with 13 experienced teachers. The results of their study demonstrated that maladaptive immunity was the dominant form of immunity among the participants. Similarly, Maghsoudi (2021) conducted a study to identify the dominant type of teacher immunity in Arak, Iran, and utilized a questionnaire and a semi-structured interview to gather the required data. Finally, the results of MAXQDA (ERBI Software, Berlin, Germany), ANOVA, and *t*-test indicated that productive (i.e., positive) immunity type was dominant among the prospective EFL teachers. It was also concluded that teacher immunity is a dynamic construct that can influence almost everything that the teachers do in their profession. In sum, the review of empirical studies on the three variables of concern in this study signifies that they still need more complementing studies in different contexts. Most of the empirical studies in this domain have taken advantage of quantitative research designs, examined only the views of teachers, and conducted them in one cultural context. These yawning gaps provided the motive for running this review article on the PWB, mindfulness, and immunity of teachers, which are interconnected constructs (Figure 2).

## Implications for Future Inquiries

In light of this review article, it was certified that emotions, affect, and psychology of teachers play a crucial role in education as they are the frontline practitioners whose performance depends on many internal and external factors. Hence, the achievement, motivation, and learning of students all depend on the psychological states of their teachers. Teaching is known as a stressful and demanding profession that places tensions and accountability pressures on teachers. So, to cope with such impositions, teachers must be psychologically prepared and trained. In this theoretical review, we presented the theoretical underpinnings of the psychology and emotion of teachers. More specifically, drawing on PP, three novel constructs related to the psychology of teachers, namely, PWB, mindfulness, and immunity were comprehensively explained referring to their definitions, conceptualizations, dimensions, underlying theories [e.g., the CDST, self-organization, the reflexive self-consciousness theory, integrative awareness theories, and the mindfulness framework of Shapiro et al. (2006)], empirical studies, and their multiple impacts on teachers and students. Based on this review, this strand of research has precious implications for different stakeholders in academic settings such as students, teachers, teacher educators, material developers, policymakers, and researchers.

Considering the EFL students, this line of research is beneficial in that it maximizes the awareness of learners regarding the co-constructed nature of language teaching and their active role in shaping the identity and instructional efficacy of teachers. When they are cognizant that the responsibility of incurring learning is not the exclusive duty of their teacher, they try to help in minimizing and even removing classroom tensions and stressors so that their teacher feels relaxed and does his/her best when teaching a subject. Similarly, the motivation, interest, and engagement of students improve in a context in which they are regarded as the owners of the learning process. As for teachers, the results would be of help in that they get familiar with the fact that language teaching is a demanding profession that is full of adversities, tensions, and traumatic experiences (Derakhshan, 2021). Correspondingly, they can provide insights about ways to cope with such adversities and disturbances through suitable strategies and provide a comfortable environment for students to learn English with pleasure. They can also offer mediation practices to students and create a mindfulness learning experience in which the motivation and interest of students upsurge.

Moreover, teacher educators can use the information provided in this review to design professional development courses for both pre- and in-service EFL teachers in which the complexities and challenges involved in the teaching profession are unraveled and appropriate protective techniques are taught. They can also devote some time and effort to develop the positive emotions of teachers along with the pedagogical aspects of teaching. Teacher trainers can improve knowledge and also application of mind and attention-related strategies of teachers. Furthermore, material developers can write materials in which the emotions of teachers are reflected and the lessons and activities help teachers in reducing their stress and pressure of the job, enjoying their moment, and cultivating a defensive mechanism in them to stand against adversities and traumatic events of teaching a foreign language. Similarly, policymakers can revisit their decisions and plans in educational contexts and provide an opportunity for teachers to feel comfortable, cared for, valued, and immunized. Teachers should not be seen only as tools to inject knowledge to learners without considering their emotions and affective needs. Finally, researchers can benefit from this study in that they can conduct more studies on similar constructs related to this research territory.

Although this line of research has yielded useful insights about the psychology of teachers and language education, it suffers from some flaws that should be solved. One of the limitations of this area is that it has mainly been explored using one-shot designs and quantitative measures although, in some studies, interviews and case studies have also been used. PWB, mindfulness, and immunity of teachers are the psychological traits that are usually formed and developed by the passage of time in dynamic processes. Nevertheless, few (if any) longitudinal and extensive explorations have been conducted on these variables which are highly recommended to future scholars. Another gap is that qualitative-oriented research studies focusing on how these constructs operate in EFL, English for specific purposes (ESP), and English for Academic purposes (EAP) contexts are non-existent. Future studies can be carried out using diary, portfolio, observation, and reflective journals to unpack the ways through which such variables affect teachers and students. Furthermore, most of the studies in this domain have employed the non-random sampling techniques that limit the generalizability scope of their conclusions (Bornstein et al., 2013). As a result, future researchers can use random sampling in experimental studies to see the effect of training these constructs on the pedagogy of teachers.

Similarly, the role of culture and context in developing and reshaping these variables has been limitedly explored. Educational contexts in secular and socialist cultures are not the same; hence, running cross-cultural studies is a fresh idea for research in this domain (Özü et al., 2017). The findings of many studies approved that the three constructs examined in this review affect almost all aspects of the career of a teacher. However, their contributions and impacts on the classroom teaching practices of teachers regarding different language skills have not been sufficiently investigated. Similarly, this line of research has mainly focused on the perspectives and operations of teachers, regarding the three variables and the viewpoints of other parties have been excluded. Therefore, future researchers can carry out analogous studies integrating the perceptions of different stakeholders in this regard. Additionally, it should be noted that among the variables of concern in this review, the wellbeing of teachers has been more explored in comparison with the mindfulness and immunity of teachers. So, avid researchers are recommended to work on these two recent constructs in relation to other variables such as self-efficacy, academic buoyancy, optimism, happiness, and interpersonal communication behaviors (e.g., clarity, credibility, and immediacy). Finally, perusing the related studies in this research territory, we can easily observe that the explorations of PWB, mindfulness, and immunity of teachers have been mostly made without taking demographic factors of the participants into consideration. Consequently, future studies are suggested to examine the mediating role of age, gender, experience, major, and academic degree on the level and development of such constructs. All these shortcomings are indicative of the fact that this line of research is still in its beginning stages and calls for more investigations.

## Author Contributions

SL drafted and finished the manuscript, and got it ready for submission.

## Conflict of Interest

The author declares that the research was conducted in the absence of any commercial or financial relationships that could be construed as a potential conflict of interest.
